# The effects of interactive reading on young children’s narrative abilities: a meta-analytic study

**DOI:** 10.3389/fpsyg.2025.1653511

**Published:** 2025-11-05

**Authors:** Lei Xing, Yi Tang, Qingke Liu, Haifeng Chen, Jiamin Zeng, Junyue Su

**Affiliations:** 1 Chongqing Preschool Education College, Chongqing, China; 2 Four-Leaf Clover Kindergarten, Chongqing, China; 3 Chongqing Early Childhood Education Quality Monitoring and Evaluation Research Center, Chongqing Normal University, Chongqing, China; 4 Xincun Kindergarten, Chongqing, China

**Keywords:** interactive reading, narrative abilities, meta-analysis, early childhood education, language education

## Abstract

**Background:**

The development of narrative abilities during early childhood forms the foundation for more complex language expression and comprehension later in life. This study employs a meta-analytic approach to systematically evaluate and infer the effects of interactive reading on young children’s narrative abilities.

**Methods:**

Inclusion criteria for eligible studies were established. Electronic databases, including CNKI, Web of Science, ScienceDirect, SpringerLink, Taylor & Francis, Wiley, and ERIC, were systematically searched for experimental or quasi-experimental studies investigating the effects of interactive reading on young children’s narrative abilities. A moderator analysis was subsequently conducted to explore potential factors influencing the effectiveness of interactive reading interventions.

**Results:**

A total of 25 studies (*k* = 123 independent effect sizes; *N* = 2,886 participants) were included. Random-effects modeling revealed significant heterogeneity (*I*^2^ = 76.07%, *p* < 0.001). Key findings: (1) Interactive reading exerted a medium aggregate effect on narrative ability development [*g* = 0.425, 95% CI (0.333, 0.518), *p* < 0.001], per Cohen’s benchmarks. (2) The effect on children’s narrative development was significantly moderated by the duration of the interactive reading intervention. (3) Incorporating peer sharing during interactive reading significantly enhanced the development of children’s narrative abilities.

**Conclusion:**

Interactive reading has a positive intervention effect on children’s narrative abilities, and this effect is influenced by multiple moderating variables. This meta-analysis provides quantitative evidence supporting the role of interactive reading in promoting the development of children’s narrative abilities. Future meta-analyses could simultaneously include both preschool and school-age children to compare and analyze the intervention effects across different age groups.

**Systematic review registration:**

doi: 10.37766/inplasy2025.10.0086, INPLASY2025100086.

## 1 Introduction

### 1.1 Narrative ability represents a crucial aspect of children’s language development

Narrative, or storytelling, is a form of language expression that occurs outside of an immediate context (Bruner, 1987), guided by an individual’s internal cognitive schema, the individual verbally recounts past events (Bouizegarene et al., 2024). It includes narrative structure, thematic relevance, narrative tone, dialogue, time marking, expressiveness, vocabulary level, and sentence structure (Farah et al., 2019). Children’s language learning is characterized by comprehension preceding expression. Narrative ability serves as a bridge between content comprehension and language expression. The development of narrative ability enables children to perceive objective realities more clearly, comprehend language content in greater depth, and internalize it effectively (Liu et al., 2011). This allows children to integrate fragmented experiences into coherent narratives, express their emotions and opinions, and enhance their cognitive abilities (Cremin et al., 2013). Children’s narration skills typically develop gradually beginning at the age of three (Filiatrault-Veilleux et al., 2016), the preschool stage represents a critical period for the development of narrative ability. Relevant studies have demonstrated that early narrative intervention facilitates children’s transition from oral to written language, playing a crucial role in the development of linguistic intelligence (Kateeb, 2018; Spencer and Petersen, 2020).

### 1.2 Research on the development of narrative ability in young children

Children’s narrative ability continues to develop with age, increasingly reflecting their personal opinions (Hibbin, 2016), enhanced fictional ability (Vretudaki and Tafa, 2022), and the use of more complex expressive elements (Frizelle et al., 2018; Otwinowska et al., 2020) are influenced by factors such as family economic status (Sabourin-Guardo et al., 2024), cultural environment (Kan et al., 2020), and parenting style (Ganotice et al., 2017). Existing scholars from the personal event narratives and fictional narratives (McCabe et al., 2008; Westby and Culatta, 2016; Lai, 2020; Mills et al., 2021) studies children’s narrative ability from two aspects: the former is children’s description of real events in the past life, while the latter focuses on children’s re-creation based on pictures or videos. Some scholars have also examined narrative ability from the perspective of narrative structure at both the macro and micro levels (Lindgren, 2023; Otwinowska et al., 2020; Lipner et al., 2024; Sheng et al., 2020). Structure is the most prominent feature of storytelling. When young children tell stories, they must engage a range of narrative skills, including the ability to produce complex sentences, use morphological grammar and vocabulary (i.e., microstructures), as well as the ability to organize grammatical components and construct coherent narratives (i.e., macrostructures) (Otwinowska et al., 2020). The microstructure of narrative ability typically emphasizes lexical diversity, syntactic complexity, and pragmatic appropriateness, whereas the macrostructure focuses on the overall logical coherence of the story. Children’s narratives are typically examined at both macro and micro levels; however, research is not limited to their story expression and sentence structure (Spencer and Petersen, 2020). In fact, children’s storytelling also reflects the development of various core skills, including neural mechanism (Romeo et al., 2018), cognitive skills (Kim S. J., 2016; Kim Y. S. G., 2016) and even social ability (Brinton and Fujiki, 2017). At present, the international narrative assessment criteria show a diversified development trend, and the following results are derived from different dimensions: The Edmonton Narrative Norms Initiative, Monitoring Indicators of Scholarly Language (Gillam et al., 2017), Narrative evaluation Protocol (Justice et al., 2010) and Colorful Spectrum Language Evaluation System (Kryiakowski, 2015). A substantial body of empirical research indicates that early interventions targeting children’s narrative abilities positively influence their listening comprehension, receptive vocabulary, and writing skills (Spencer and Petersen, 2020), but also serves as a significant predictor of their academic achievement in both primary and junior high school (Xuan, 2007; Yang et al., 2020). Therefore, many countries have regarded the development of narrative ability as a key focus in the study of children’s overall skill development.

### 1.3 Research on interactive reading narratives

Interactive reading is the act of sharing or reading books with children in a relaxed and pleasant atmosphere (Noble et al., 2019). Reading is a process in which children engage with others, with the interactors stimulating children’s thinking and reflection through questions, discussions, and role-playing (Doyle and Bramwell, 2006). Unlike traditional one-way reading, interactive reading emphasizes the development of positive interactive relationships throughout the reading process (Dixon-Krauss et al., 2010; Balog et al., 2024). Children freely share their interpretations of the text with adults or peers, review the material in various forms according to their preferences, and engage in active, equitable communication with the other participants, using language to construct their cognitive frameworks (Li, 2020).

According to cognitive load theory, individuals must process and transform unfamiliar information during reading tasks. Exposure to rich and diverse linguistic input in dialogues enables children to acquire and imitate complex sentence structures and vocabulary, thereby enhancing their expressive language abilities. Consequently, interactive reading serves to construct scaffolding for comprehension and facilitates language production in children (Dillon and Newman, 2023; Wall et al., 2022). Research has demonstrated that interactive reading promotes the development of various early language skills in young children, including phonological awareness, vocabulary acquisition, and narrative competence. As a result, early reading interactions are widely recognized as crucial for fostering children’s narrative development (Li, 2020; Vretudaki, 2022). However, due to limitations in research scale and longitudinal tracking, studies on interactive reading face significant challenges, including insufficient empirical evidence and limited generalizability.

### 1.4 Ongoing debates persist regarding the effects of interactive reading on the development of children’s narrative abilities

Since the emergence of research on children’s narrative abilities, interactive reading has commonly been employed as an intervention to support narrative development. Scholars have highlighted its significant role in enhancing children’s narrative recall, production, and structural organization. The question-and-answer format of interactive reading is particularly effective in helping preschoolers navigate the “leapfrog period” of narrative development (Lai et al., 2010). Although interactive reading demonstrates considerable potential in enhancing young children’s narrative skills, researchers continue to face several controversies and challenges. First, the effectiveness of interactive reading as an intervention remains debated. Some scholars argue that interaction with adults during reading may, in certain cases, increase young children’s cognitive load, thereby hindering comprehension and narrative development (Jimenez and Saylor, 2017; Read et al., 2023), this may result in ineffective interventions. On the other hand, other studies suggest that sustained dialogue and extended interaction with others are positively associated with improvements in children’s narrative abilities (Xu, 2017; Yang et al., 2024).

Second, individual differences among young children in narrative development present challenges for making horizontal comparisons across interactive reading experiments. Considering variations in cognitive abilities, language expression, and cultural backgrounds, the effects of interactive reading interventions on children’s narrative skills are often complex and multifaceted (Barone et al., 2019; Rochanavibhata and Marian, 2021). Therefore, it is necessary to integrate a large number of different types of experimental evidence to support the research of interactive reading on children’s narrative ability.

Finally, empirical evidence regarding the long-term effects of interactive reading on children’s narrative abilities remains limited (Barone et al., 2019). Due to limitations in research scale, current intervention studies on interactive reading often lack longitudinal tracking. Even in long-term studies, some aspects of young children’s narrative skills show minimal or non-significant improvement over time (Dias-Broens and van Steensel, 2023).

### 1.5 Age, object of interaction, and moderating variables of intervention duration

Relevant studies have pointed out that children’s narrative development presents phased characteristics, and age is a key factor in the development of their narrative ability (Yang et al., 2020). For some younger children, the narrative task of requiring them to generate a complete story without any oral input is particularly difficult, which makes researchers doubt whether the narrative generation task for young children effectively triggers certain types of complex grammar (Frizelle et al., 2018). Some scholars also point out that the key to ensuring the quality of interactive reading is to wait appropriately for the development of children’s language ability (Khan et al., 2016). Therefore, the selection of children at which age to intervene has become a key problem in today’s narrative intervention.

The interactive relationship has a significant impact on the quality of interactive reading. In recent years, scholars have been exploring the positive influence of parents, teachers and peers on the intervention of children’s narrative ability (Sang, 2018; Lingwood et al., 2020), but a comparison of corresponding effects is lacking. In view of this, this study chooses interactive objects as the moderating variable for discussion and divides them into peers, teachers, parents and researchers.

Intervention duration is also an important variable that affects the narrative development of young children, and the contrast between long-term effect and short-term effect is a contradictory problem in current interactive reading intervention. Some scholars have explored the impact of interactive reading on children’s narrative development through long-term intervention (Silva and Cain, 2024), and some scholars obtained immediate results through short-term intervention (Yang and Zheng, 2020; Riad et al., 2024). In view of this, intervention duration was selected as the moderating variable for discussion in this study, and intervention duration was divided into less than 8 weeks, 9–16 weeks, and more than 17 weeks.

### 1.6 Purpose of this study

Experimental studies related to this topic are screened out, disputes existing in existing literature are discussed, and the regulating effects of children’s age, interactive objects and intervention duration are further analyzed. Therefore, according to existing research contents, this study mainly discusses the following questions:

1) Evaluate the overall effect of interactive reading on the intervention effect of children’s narrative ability, whether interactive reading can promote the development of children’s narrative ability, and if so, what is the extent of its influence.
2) Age adjustment: Whether there are differences in the development of narrative ability of children of different ages in interactive reading, and if so, which age group can improve their mental health more.
3) Adjustment of interactive objects: whether different interactive reading objects have differentiated effects on the development of children’s narrative ability, and if so, which interactive objects have the greatest impact.
4) Adjustment of intervention duration: whether there are differences in the intervention effects of interactive reading on children’s narrative ability under different intervention duration.

## 2 Methods

### 2.1 Information sources and search strategy

**Figure 1:**
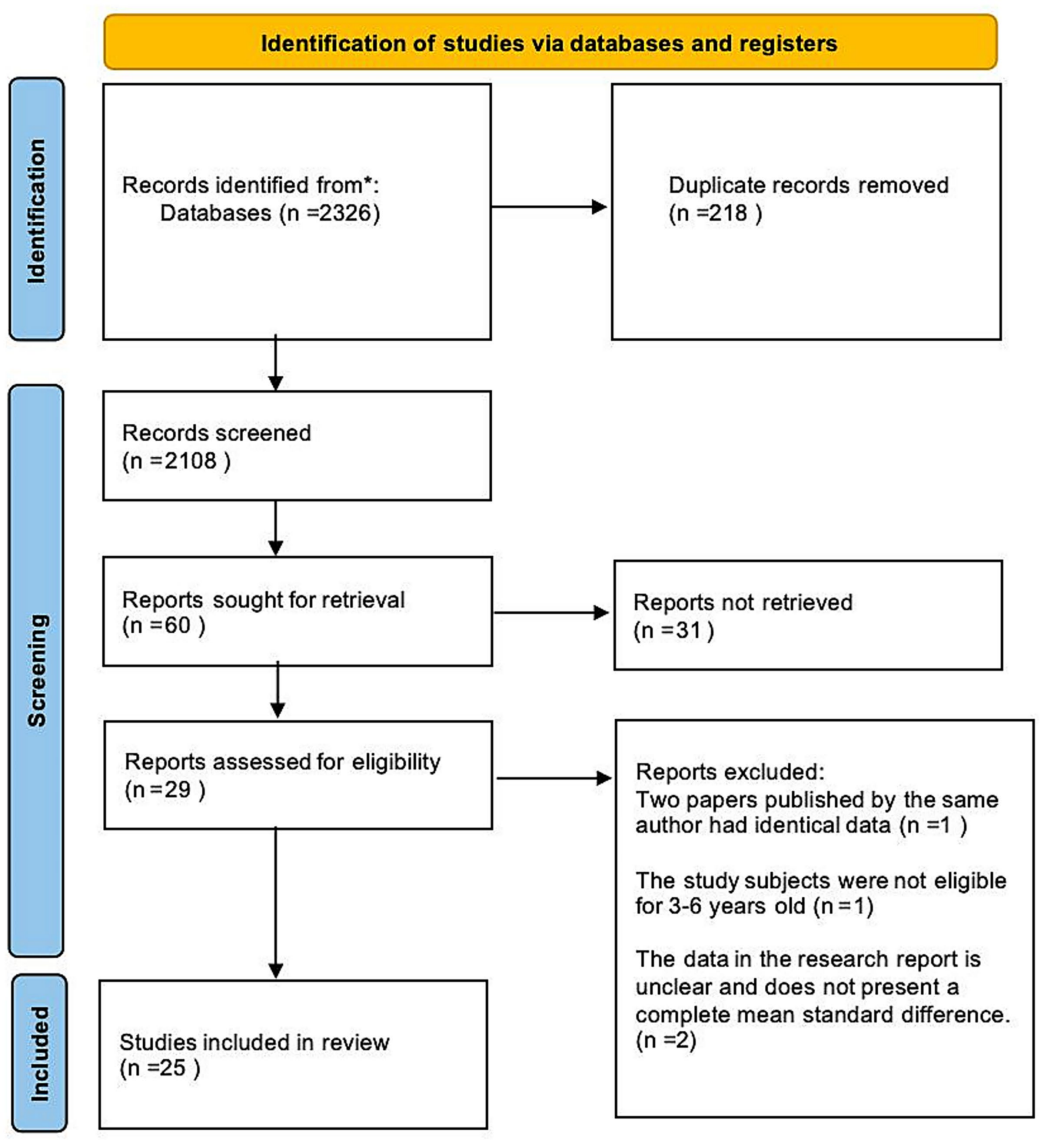
PRISMA flowchart.

Systematic review and meta-analysis were carried out in accordance with the PRISMA guidelines. Chinese literature was retrieved from CNKI, while English literature was sourced from the Web of Science, Science Direct, SpringerLink, Taylor & Francis, Wiley, and the ERIC electronic databases. At the same time, the method of literature backtracking was used for literature supplementary search. Three sets of keywords were used to search: (1) [“Young children” OR “preschoolers” OR “infants” OR “toddlers” OR “Child” OR “children”]; (2) [“shared book reading (SBR) “OR “shared reading” OR “interactive reading” OR “dialogic reading” OR “conversational reading”]; (3) [“narrative ability” OR “Oral Narrative” OR “Storytelling” OR “Narrative Skills” OR “Oral Narrative” OR “Narrative Skills”]. The two coders searched a total of 2,326 relevant studies in the database, deleted 218 duplicate data, and left 2,108 references. After reading the literature in strict accordance with the inclusion criteria, 2079 literatures were excluded, leaving 29 qualified reports. Among the 29 studies, after careful reading again, one study with the same data published by the same author was excluded, one study whose subjects were not between the ages of 3–6 years old was excluded, and two studies with unclear data and incomplete mean and standard deviation were excluded. Finally, the remaining 25 studies were included in the analysis (see Figure 1).

### 2.2 Inclusion criteria

The criteria for literature inclusion in this meta-analysis were:

1) The literature was an experimental or quasi-experimental study, randomly assigned to the experimental group and the control group to receive the intervention.
2) Complete indicators such as mean and standard difference of the experimental group and the control group were clearly reported, and the data were complete so as to calculate the effect size.
3) The study subjects were children aged 3–6 years without any cognitive, language or physical disabilities.
4) The topic was the impact of interactive reading on narrative ability.
5) If there is data duplication in two papers published by the same author, select data from only one paper.

### 2.3 Data coding

Two authors extracted the following information from the included literature: (1) author, year of publication, and country; (2) subjects of study; (3) sample size (experimental group/control group); (4) Intervention details of experimental group and control group (interaction subjects, intervention duration); and (5) data for effect size calculation.

### 2.4 Statistical analysis

In this study, Stata17.0 software was used for meta-analysis, Hedges’ *g* was used as the effect size, forest map, heterogeneity test, publication bias and other functions in the meta-analysis menu were used for analysis, and random effects model was selected. This study was composed of 25 interactive reading experiments with 123 effect sizes to explore the effects of interactive reading on children’s narrative ability at different ages, different intervention duration and different interactive objects.

### 2.5 Publication bias test

**Figure 2:**
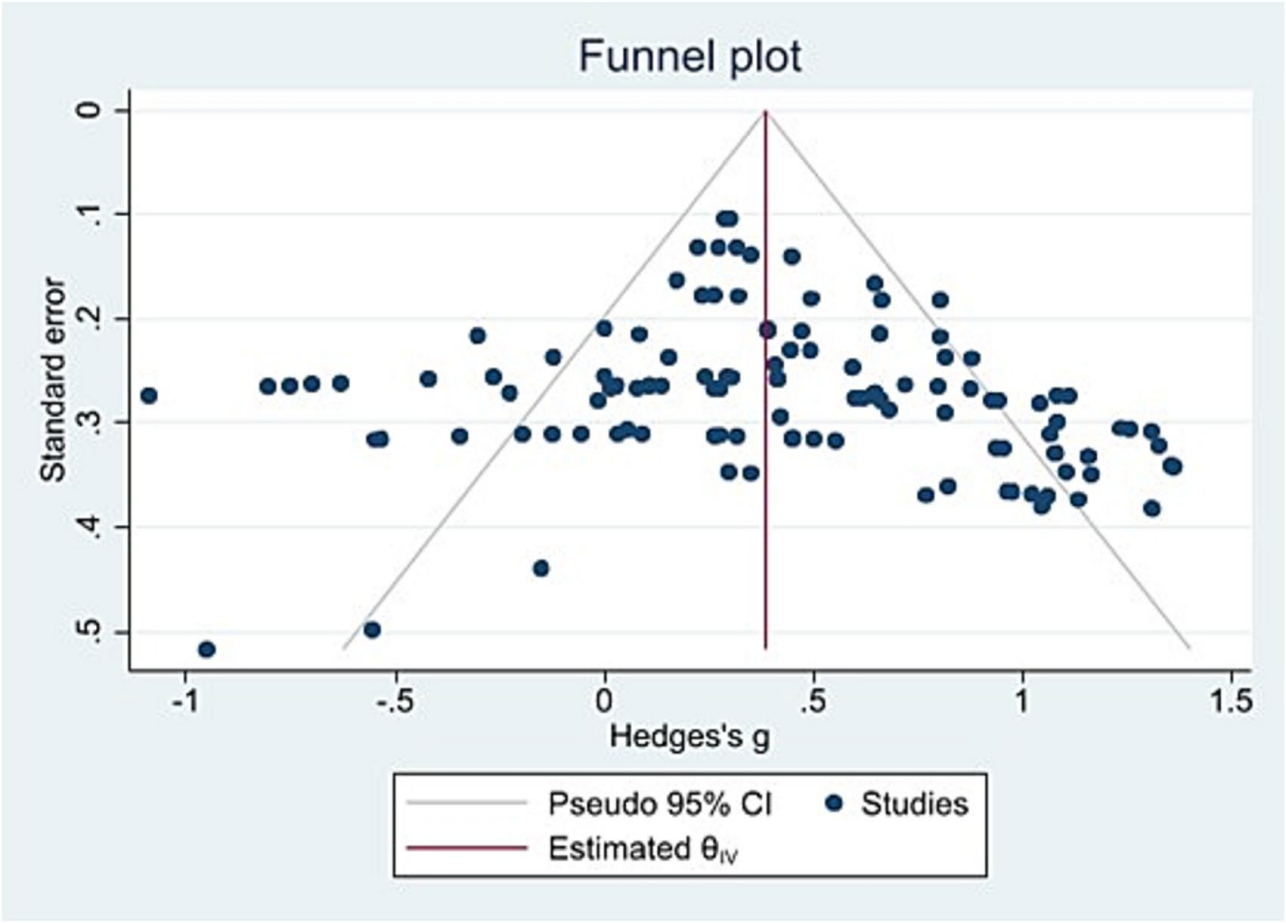
Publication bias funnel plot.

Publication bias refers to the fact that the results of “statistically significant” positive studies in existing studies are more likely to be published than those of “statistically significant” negative studies, resulting in a bias in the results of meta-analysis. The funnel plot method and Egger method were used in this study to test for publication bias. In the detection of funnel plot method, if there is no publication bias, the scatter points of funnel plot will be symmetrically distributed around the true value and tend to be concentrated in a narrow range. From the funnel plot of this study, as shown in Figure 2, the scatter-point distribution of the effect values of independent studies included in the study had no obvious asymmetry, and most of them were in the middle region of the funnel plot and relatively evenly distributed on both sides of the median line, indicating that the possibility of publication bias was small. At the same time, the Egger linear regression test results show that the *t* value is 1.76 and the *p*-value is 0.0803 (*p* > 0.05), indicating that there is no publication bias in this study and the meta-analysis results are relatively stable and reliable.

### 2.6 Test of heterogeneity

**Figure 3:**
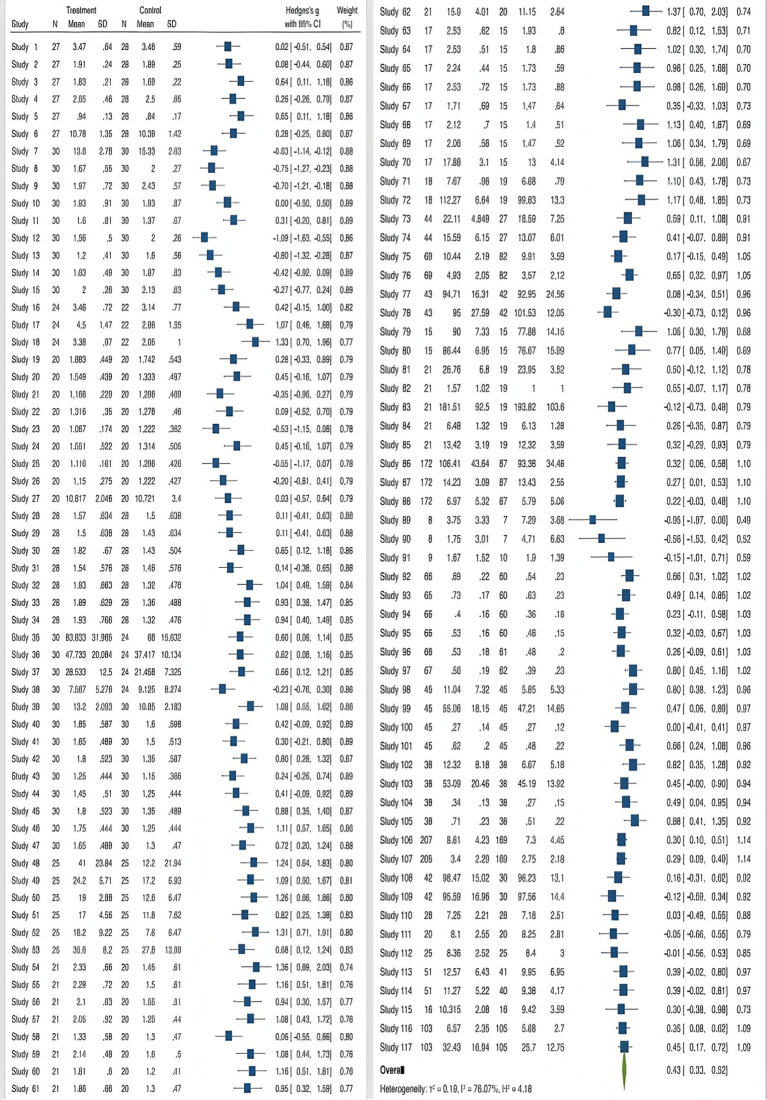
Forest diagram.

When using the meta-analysis method to analyze the sample literature, attention should be paid to the heterogeneity of the findings and publication bias. Since various studies included in the same systematic review in the meta-analysis may have random sampling error and variation between study groups, resulting in heterogeneity of the effect size between studies, the heterogeneity test method must be adopted for evaluation. The *Q*-test combined with the *I*^2^ statistic is the best scheme to test heterogeneity. Generally speaking, *I*^2^ = 25, 50, 75% is used to divide heterogeneity into three levels: low, medium and high. In this study, the *Q* test combined with the *I*^2^ statistic was used to test the heterogeneity of the sample literature data. The *Q* value of the test result was 399.50 (*p* < 0.001), and the *I*^2^ value was 76.07% (greater than 75%), indicating that the study had high heterogeneity, and two effect models could be selected to eliminate the influence of heterogeneity. Random effect model and fixed effect model. A random effects model can be selected if heterogeneity is high, and a fixed effects model can be selected if heterogeneity is low. Therefore, according to the test results, this study will adopt the random effects model for analysis, and then evaluate the effect of interactive reading on children’s narrative ability (see Figure 3).

## 3 Results

### 3.1 Bias assessment

**Figure 4:**
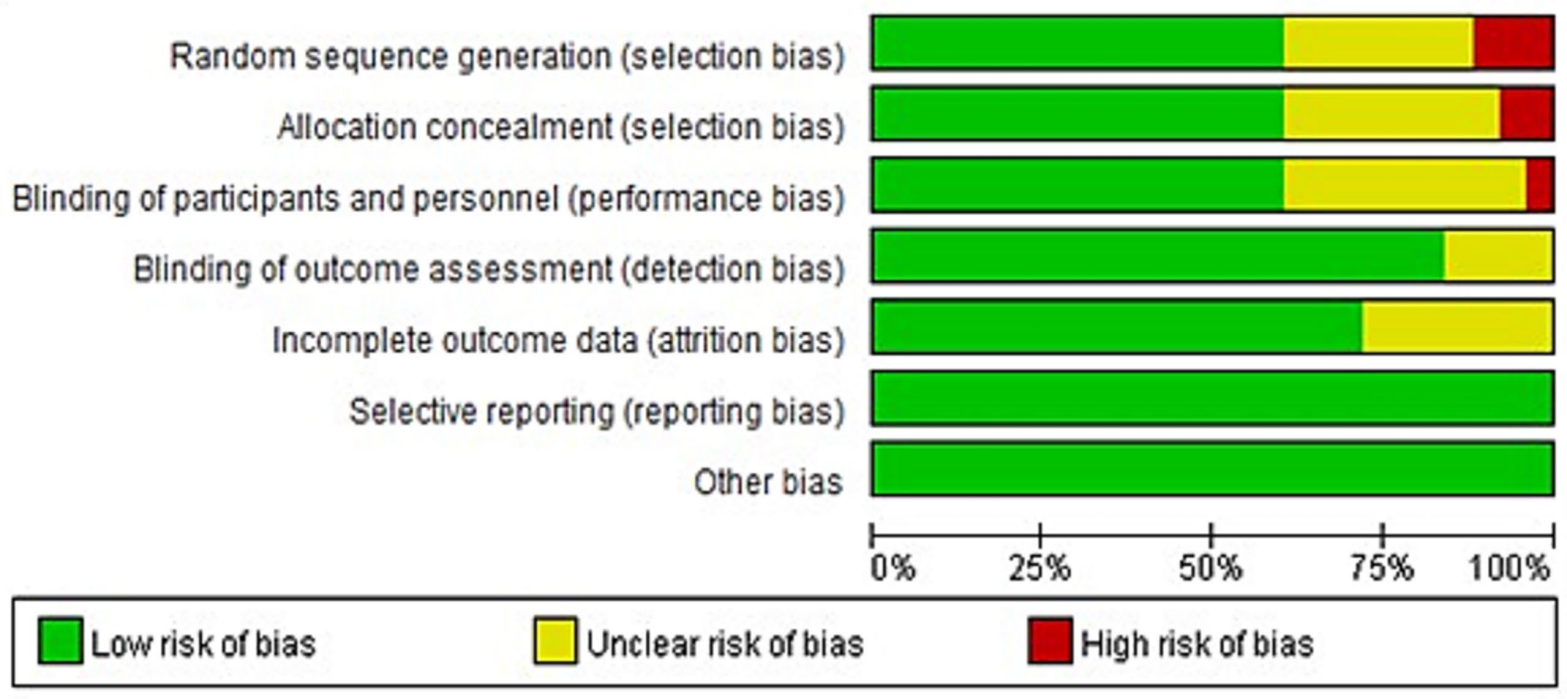
Publication bias quality assessment.

The Cochrane risk of bias assessment tool in Revman software was used to evaluate the quality of the literature included in the meta-analysis, mainly from six areas, including selection bias, measurement bias, follow-up bias, reporting bias, implementation bias and other bias. For each indicator, high risk of bias, low risk of bias and uncertainty of bias were used to evaluate, as shown in Figure 4. If all the literatures are low-risk in the evaluation process, the quality grade of the literatures is grade A, and the possibility of bias is the least. If the risk is unknown in the process of literature evaluation, the quality grade of the paper is B, and there is a medium possibility of bias. In the evaluation process, as long as one item is high risk, the quality of the paper is level C, with a high possibility of bias (Liao et al., 2023). According to the results of bias risk assessment, there were 7 articles with grade A quality, 15 articles with grade B quality, and 3 articles with grade C quality. Among them, 10 literatures were not rigorous enough in randomization, could not specify the way of randomization, or simply grouped children according to the order of participation; In 10 papers, there were loopholes in allocation and hiding. The literature lacked descriptions of whether the groups were hidden or not, or there was the possibility that children, trainers and researchers could know the grouping situation. In 9 literatures, it was not possible to determine whether blind or incomplete blind method was implemented. In 3 literatures, there was the possibility of incomplete outcome data in the scale scoring of children; and 7 articles were uncertain about the existence of incomplete outcome data. Overall, the overall level of literature quality was good.

### 3.2 Overall impact effect test

Cohen’s effect size benchmarks classify values under 0.2 as negligible, those between 0.2–0.5 as moderate, and exceeding 0.5 as substantial. Table 1 reveals an effect magnitude of 0.425, positioned within the moderate interval (0.2–0.5). This evidences that interactive reading contributes moderately yet positively to the holistic progression of children’s narrative capabilities.

**Table 1:** The overall effect of interactive reading on children’s narrative ability.

Effect model	*n*	*g*	95% CI		Heterogeneity test			
			Lower limit	Upper limit	*I*^2^ (%)	*Q*	df	*p*
Random effects model	117	0.425	0.333	0.518	76.07	399.50	116	0.000

### 3.3 Test of moderating effects

#### 3.3.1 Age

Building upon the established moderate effect of interactive reading on narrative development, this investigation extends to examine age-specific manifestations across three ontogenetic stages (3–4 years, 4–5 years, 5–6 years). As delineated in Table 2, significant enhancements in narrative competence emerge universally (aggregate *g* > 0, *p* < 0.01), confirming developmental pervasiveness. Crucially, the 4–5 years cohort exhibits maximal effect magnitude (*g* = 0.635, *p* < 0.001), signifying a critical ontogenetic window for intervention efficacy.

**Table 2:** Test of the moderating effect of interactive reading on narrative ability of children at different ages.

			95% CI		*p*	Heterogeneity test	
Age	*n*	*g*	Lower limit	Upper limit		*Q*	*I*^2^ (%)
3–4 years	8	0.303	0.127	0.478	0.001	11.96	40.38
4–5 years	50	0.635	0.504	0.766	0.000	141.86	67.06
5–6 years	59	0.274	0.143	0.406	0.000	212.70	76.90

#### 3.3.2 Interactive objects

Table 3 demonstrates significant facilitative effects of all four pedagogical agents on children’s narrative competence (*g* > 0, *p* < 0.01). Effect magnitude stratification reveals peer interaction as the most potent predictor [*g* = 0.675, 95% CI (0.519, 0.830)], exceeding Cohen’s large-effect threshold (0.5–1.0). Subsequent predictors include parental engagement (*g* = 0.597) and researcher facilitation (*g* = 0.459), with teacher-guided interaction demonstrating modest efficacy (*g* = 0.164). This hierarchy suggests that peer-mediated linguistic scaffolding—characterized by reciprocal discourse patterns, observational learning mechanisms, and low-affect communication contexts—optimally catalyzes narrative development. While parental, researcher, and pedagogical inputs remain valuable, their differential efficacy necessitates strategically diversified interventions prioritizing peer dyads and family participation.

**Table 3:** Test of the moderating effect of interactive objects on children’s narrative ability under interactive reading.

			95% CI		*p*	Heterogeneity test	
Object of interaction	*n*	*g*	Lower limit	Upper limit		*Q*	*I*^2^ (%)
Companion	26	0.675	0.519	0.830	0.000	54.10	53.85
Teachers	44	0.164	0.012	0.316	0.034	163.83	79.59
Parents	28	0.597	0.386	0.808	0.000	91.48	73.97
Researcher	19	0.459	0.335	0.584	0.000	28.47	33.04

#### 3.3.3 Duration of intervention

To investigate temporal dynamics, this study examined duration-dependent effects of interactive reading interventions. Table 4 reveals a monotonic dose–response relationship: effect magnitudes escalate with intervention length, peaking at ≥17 weeks [*g* = 0.644, 95% CI (0.485, 0.803)], followed by 9–16 weeks [*g* = 0.484, 95% CI (0.369, 0.599)], and ≤8 weeks [*g* = 0.267, 95% CI (0.120, 0.413)]. Critically: (1) All durations exceed Cohen’s small-effect threshold (*g* > 0.20), confirming universal efficacy. (2) ≥17-week interventions reach large-effect magnitude (*g* > 0.50). (3) Significant between-group gradients exist (Δ*g* max-min = 0.377, *p* < 0.001). These findings establish temporal accumulation as a key efficacy modulator, where extended implementation generates cascading gains in narrative competence through sustained linguistic scaffolding.

**Table 4:** Test of the moderating effect of intervention duration on children’s narrative ability under interactive reading.

			95% CI		*p*	Heterogeneity test	
Duration of intervention	*n*	*g*	Lower limit	Upper limit		*Q*	*I*^2^ (%)
17 weeks and older	30	0.644	0.485	0.803	0.000	76.42	65.71
8 weeks and under	58	0.267	0.120	0.413	0.000	239.31	77.06
9–16 weeks	29	0.484	0.369	0.599	0.000	53.64	50.95

## 4 Discussion

### 4.1 The overall effect of interactive reading on children’s narrative ability

Statistical analysis of 25 relevant documents shows that interactive reading has a moderate positive effect on the development of children’s narrative ability (see Table 5). This research result is confirmed by several previous studies (Reese et al., 2010; Xu, 2017), to a certain extent, affirms the positive role of interactive reading as an intervention.

**Table 5:** Studies included in the meta-analysis.

Author (year)	Study	*N* (experimental group/control group)	Age	Interactive object	Duration of intervention	Intervention type
Yang and Zheng (2020)	1	27/28	4–5 years	Teachers	8 weeks and under	Group experiments in schools
Zhang (2017)	2	30/30	5–6 years	Teachers	8 weeks and under	Group experiments in schools
Xu (2017)	3	24/22	4–5 years	Researcher	8 weeks and under	Group experiments in schools
Song (2011)	4	20/20	5–6 years	Teachers	8 weeks and under	Conduct one-to-one experiments in schools
Huang (2020)	5	28/28	4–5 years	Companion	9–16 weeks	Group experiments in schools
Sang (2018)	6	30/24	5–6 years	Companion	9–16 weeks	Conduct one-to-one experiments in schools
Wang and Xie (2022)	7	30/30	4–5 years	Companion	9–16 weeks	Group experiments in schools
Li C. (2014) and Li J. (2014)	8	25/25	4–5 years	Companion	8 weeks and under	Group experiments in schools
Wu and Wu (2017)	9	21/20	4–5 years	Parents	17 weeks and older	Conduct one-to-one experiments in schools
Şimşek and Işıkoğlu Erdoğan (2021)	10	18/19	4–5 years	Teachers	8 weeks and under	Group experiments in schools
Teepe et al. (2017)	11	44/27	3–4 years	Parents	8 weeks and under	Conduct one-to-one experiments in schools
Silva and Cain (2024)	12	69/82	5–6 years	Researcher	17 weeks and older	Group experiments in schools
Lingwood et al. (2020)	13	43/42	3–4 years	Parents	8 weeks and under	Group experiments in libraries
Nevo and Vaknin-Nusbaum (2018)	14	15/15	5–6 years	Teachers	9–16 weeks	Group experiments in schools
Lever and Sénéchal (2011)	15	21/19	5–6 years	Researcher	8 weeks and under	Group experiments in schools
Thomas et al. (2019)	16	172/87	5–6 years	Teachers	9–16 weeks	Group experiments in schools
Reese et al. (2010)	17	8/7	4–5 years	Parents	17 weeks and older	Conduct one-on-one experiments in families
Grolig et al. (2020)	18	66/60	5–6 years	Researcher	17 weeks and older	Group experiments in schools
Riad et al. (2024)	19	45/45	5–6 years	Teachers	8 weeks and under	Group experiments in schools
Grøver et al. (2020)	20	207/169	4–5 years	Teachers	9–16 weeks	Group experiments in schools
Requa et al. (2022)	21	42/30	4–5 years	Parents	8 weeks and under	Conduct one-on-one experiments in families
van der Wilt et al. (2019)	22	28/28	5–6 years	Teachers	8 weeks and under	Group experiments in schools
Lake and Evangelou (2019)	23	51/41	3–4 years	Researcher	9–16 weeks	Group experiments in schools
Farah et al. (2019)	24	16/16	4–5 years	Researcher	8 weeks and under	Group experiments in schools
Burgoyne et al. (2018)	25	103/105	3–4 years	Parents	17 weeks and older	Conduct one-on-one experiments in families

There is a strong link between interactive reading and children’s narrative ability, and many researchers have explained the mechanisms of its influence from different disciplinary perspectives. Neuroscience research has shown that children show remarkable plasticity in language learning. The plasticity of the brain to language is a statistics-based process (Kuhl, 2011) in which young children build up a stable knowledge of the sounds to which they are exposed by counting them. The process of interactive reading is children’s synesthesia of multiple sensory experiences to support language processing, and they synchronize the activities within and between the brain (Thiede, 2019). During this process, the language regions of the child’s brain (such as Broca and Wernicke) become more active and stimulate speech production and comprehension in the child. Social interactionism believes that interactive reading provides children with language materials (Dowdall et al., 2020), wherein the dialogue and discussion between parents or teachers and children not only increase the diversity of stimulus input, but also help children sort out reading information and construct understanding framework through questioning, feedback and repetition. Effectively trigger the narrative and expression of young children. The development of children’s narrative skills follows a progressive pattern. With the dynamic evolution of children’s age growth and cognitive development, their ability to understand and retell stories shows a significant improvement trend. Therefore, developmental psychologists believe that questioning and predictive activities in interactive reading can effectively promote children’s metacognitive ability (van Kraayenoord, 2010), the understanding and control of their own cognitive processes. Studies have confirmed that interactive reading, as an intervention method, can effectively promote the improvement of children’s narrative ability. Specifically, with the cyclic deepening of cognitive activities such as situational reasoning and role empathy in conversational reading between adult guides and children, young children gradually achieve the cognitive leap from story understanding to retelling. This development trajectory not only reflects the dynamic balance of the “assimilation-adaptation” mechanism in Piaget’s cognitive development theory, but also echoes the core viewpoint of Vygotsky’s socio-cultural theory on the internalization of advanced psychological functions.

### 4.2 Analysis of moderating variables between interactive reading and children’s narrative ability

#### 4.2.1 The moderating effect of children’s age is significant

The results of this study show that there are significant differences in the influence of interactive reading on the development of narrative ability of children at different ages. Compared with 3–4 years old and 5–6 years old, in 4–5 years old, interactive reading has a significant effect on the development of narrative ability of children, and the greatest impact (*g* = 0.635, *p* < 0.01). This research result is consistent with the research results (Wu, 2014), interactive sharing reading intervention significantly improves the reading interest and narrative ability of 4–6 year old children. The same results were obtained in the study of Huang (2020), who found that after interactive picture book sharing reading activities, children aged 4–5 have a rich vocabulary and their vocabulary utilization level has been greatly improved, which indicates that picture book reading activities, as one of the interactive reading methods, can significantly improve the narrative ability of children aged 4–5. The above results can be explained in the following aspects:

First, Senechal et al. (2008) showed that interactive reading can promote children’s expressive vocabulary, morphology and syntax comprehension. The study of Huang (2020) found that the key stage of children’s language development, especially the 4–5 years old period, is regarded as the active period of rapid growth of vocabulary. However, vocabulary is the material of narration for children. Children with higher vocabulary level are more likely to use “building materials” to express their ideas with more words and confidence to express boldly, so they are more likely to carry out longer narration with richer content (Li C., 2014; Li J., 2014). Morgan and Meier (2008) proposed that at this critical stage, interactive reading builds a dynamic language environment through two-way dialogue between adults and young children, and has become an effective way to improve children’s narrative ability. During this process, adults use diverse vocabulary and abstract expressions. Through interactive communication and listening to feedback, they not only enhance children’s understanding and memory of vocabulary but also significantly increase their vocabulary. With the improvement of language skills, young children gradually construct narrative frameworks that are more complex in structure and more rigorous in logic, making the story content more rich and three-dimensional, and the expression of emotions more delicate and profound. In their research, German and Simon (1991) analyzed children’s word-finding skills in discourse and found that although children with word-finding disorder had no difference with normal children in language productivity, they showed more word-finding features in their narration. This indicates that the improvement of vocabulary ability can reduce the stuttering phenomenon in narration, making the narrative more smooth and the expression of emotions more delicate. Therefore, interactive reading, through its unique interactive mode, significantly promotes the rapid growth of vocabulary and the improvement of application ability of children aged 4–5, providing a solid language foundation and rich expression resources for the leap of children’s narrative ability.

Second, according to Piaget’s cognitive development theory, children aged 4 to 5 are at the end of the preoperational stage, and their cognitive development and social and emotional needs show a double leap: at this stage, children’s social needs surge, they start to build complex social skills, and they exhibit a strong desire for self-expression and interpersonal understanding. Interactive reading provides an excellent carrier for social learning during this critical period by creating in-depth dialogue scenarios between adults and children, and promotes the systematic development of children’s social interaction abilities in language interaction. For example, through story telling, young children can practice and learn how to communicate and collaborate with others in simulated social situations (Koivula et al., 2020; Kohm et al., 2016). In addition, interactive reading provides a platform for young children to express their thoughts and feelings through storytelling methods that can effectively support young children’s social–emotional reasoning, to meet their needs for social interaction and emotional development (Koivula et al., 2020). Specifically, on the one hand, social interaction provides a rich language and emotional communication environment for young children, which is crucial for the development of narrative skills. In social interaction, children learn how to organize language, express emotion and construct story framework through imitation, interaction and cooperation (Hebert-Myers et al., 2006). On the other hand, the satisfaction of emotional needs contributes to the development of children’s emotional regulation ability, which is an integral part of narrative skills. Good emotional regulation enables young children to better understand and express complex emotional states, which is essential for emotional depth and richness in narrative (Brinton and Fujiki, 2017). For example, through storytelling activities, young children learn not only how to express their own emotions, but also how to understand those of others, and this ability is an important foundation for the development of narrative skills (Betawi, 2014).

Third, the brains of 4 to 5 year olds are undergoing rapid development and reorganization, especially in brain regions associated with language processing. According to functional magnetic resonance imaging (fMRI) and other neuroimaging studies, developmental changes occur in the support networks of brain regions during the development of language skills in children from infancy to adulthood (Vannest et al., 2009). In particular, the brain’s ability to process language is developing rapidly during 4–6 years of age. Ríos-López et al. (2020) in their study recorded the development of neural oscillatory activity in response to language in children aged 4–5 years using functional magnetic resonance imaging (fMRI). This study found that from 4–6 years of age, children’s brains begin to process verbal information more efficiently, especially in processing slower-temporal components of speech, such as syllabic and prosodic information, and that delta and theta band activity in the right hemisphere is indirectly correlated with intelligibility of speech. This finding is also supported by Weiss et al. (2018) study, which examined brain processing of speech in children aged 5–6 years by functional magnetic resonance imaging (fMRI) and found that the brains of these children have shown specialization in both speech and semantic processing. This suggests that at 4–5 years of age, children’s brain language regions have begun to develop rapidly and specialize. Neural network maturation in language areas is shown by enhanced functional connectivity in children aged 4–5 years, while functional connectivity in infancy and early childhood is prospectively associated with language and basic literacy skills at 6.5 years of age (Yu et al., 2022). This suggests that the neural network connections formed in the early stage may lay a neural foundation for the subsequent development of language processing capabilities. Interactive reading may support the development of narrative comprehension by promoting effective connections between brain regions. Schmithorst et al. (2007) found that feedback networks in the brain involve efficient connections from Broca’s area and the medial side of the upper frontal lobe to the posterior part of the bilateral superior temporal gyrus when performing narrative processing tasks, and that this connectivity strengthens with age. This suggests that through interactive reading, connections between key areas of the brain responsible for language and narrative processing can be promoted, thereby supporting the development of narrative comprehension in children. Paranawithana et al. (2023) used functional near infrared spectroscopy (fNIRS) in their study to find that normal hearing infants had significantly enhanced functional connections between primary language areas during their first year of life. This enhanced functional connectivity helps improve the efficiency and complexity of language processing, which in turn supports more complex language tasks such as narrative.

#### 4.2.2 Interactive object analysis in interactivity

Through a systematic review of 24 literatures, the research results show that there are significant differences in the effect of different interactive objects on promoting the development of children’s narrative ability. In interactive reading, teachers, peers, parents and researchers have different effects on children’s narrative ability. Among them, the influence of peers was the most significant (*g* = 0.675, *p* < 0.01). Then came parents (*g* = 0.597, *p* < 0.01), then researchers (*g* = 0.459, *p* < 0.01), and finally teachers (*g* = 0.164, *p* < 0.01). A large number of research evidences show that peer influence is more obvious in interactive reading’s impact on children’s narrative ability. Among them, Sang (2018) found in her study that peer sharing reading has a more significant impact on all dimensions of children’s narrative ability than independent reading. The results of this study indicate that peer sharing reading activities can be used as an effective method and means to cultivate the development of children’s narrative ability in kindergartens. Bokus (1992) found that compared with narration alone, children can introduce new reference content and operate on the partner’s text, such as confirmation and supplement, when they narrate with their partner. This suggests that peer interaction can enrich narrative content and structure and promote the development of narrative skills. Wei (2004) found that stories written in groups have advantages in terms of length, richness of detail, use of characters, and the use of higher levels of connectives and character representations compared to stories written individually. This suggests the potential advantages of peer cooperation in promoting young children’s narrative ability. The above results can be explained in the following aspects:

First, peer interaction is not limited to language communication, but also includes the understanding and expansion of the story content (Kim S. J., 2016; Kim Y. S. G., 2016). Research points out that peer relationships and interaction play an important role in bilingual children’s response to picture books, and these interactions affect their literary response. This suggests that peer involvement can enrich young children’s narrative experience and enable them to understand and construct stories from different perspectives.

Second, the naturalness and authenticity of social interaction. The social advantage of peer interaction is reflected in its natural characteristics of the interaction field: Children at the end of the preoperational stage can achieve stress-free self-expression in peer communication. This de-authoritative communication mode is more in line with the critical period needs of the development of their social interaction skills. This free expression helps young children to better develop narrative skills because they can explore and expand their storytelling without adult intervention (Laird et al., 1994).

Third, diversified modes of communication. Peer interaction creates diversified interactive scenarios covering dimensions such as dialogue and consultation, viewpoint confrontation, and role immersion These diverse modes of communication can help children learn how to organize language, construct plots, and express emotions, which are important components of narrative skills (Laird et al., 1994).

Fourthly, role switching and perspective switching in peer interactions help young children better understand and apply different narrative strategies. Bokus (1992) showed that in peer cooperation, children not only create stories together, but also complement and confirm each other’s narratives. This interactive process helps them learn how to view problems from different perspectives and use more complex connective words and role performance in narrative. This multi-perspective narrative training is essential for the development of children’s narrative skills.

Fifth, Peer interaction builds a unique social support system, providing immediate feedback and emotional support for the narrative process. This immediate feedback mechanism prompts young children to continuously optimize narrative expression during interaction, significantly enhancing narrative quality and content depth (Wang and Cassell, 2003). In contrast, teachers and parents, while also able to provide support and feedback, are often more fixed and authoritative in their roles and may not be as natural and varied as feedback in peer interactions.

Furthermore, when interpreting the universality of the conclusions of this study, it is necessary to take into account the specific context in which they are based. The vast majority of the empirical studies included in this review were conducted in school Settings. This distribution feature provides particularly solid evidence to support our finding that peer interaction in interactive reading can effectively drive the development of narrative ability in young children in educational contexts. However, this also means that the direct evidence that this study can provide regarding the role of peer interaction in the family (usually dominated by parents), libraries or other informal educational Settings is relatively limited. Due to the insufficient number of studies in these contexts, we were unable to conduct effective subgroup analyses to examine the moderating effects of the “context” factor. Therefore, the core conclusion of this study is first and most directly applicable to school education practice. We call for future research to be extended to more diverse social and cultural contexts to verify the robustness of current findings and to deeply reveal the possible contextual specificity of interactive reading mechanisms. This will help provide more targeted theoretical guidance for peer interaction reading intervention in different scenarios.

#### 4.2.3 Duration of intervention

Studies show that the duration of interactive reading is significantly positively correlated with the development of children’s narrative ability. With the extension of the intervention time, the amount and complexity of language input that children are exposed to gradually increase. This progressive stimulation can not only strengthen the language foundation, but also promote their understanding and application of complex narrative structures. This finding is consistent with the findings of Mol et al. (2008), which showed that even short-term interactive reading interventions can have a positive impact on young children’s language and narrative skills. Peterson et al. (1999) also mentioned in their study that long-term interactive reading, such as 1 year of intervention, can significantly improve children’s vocabulary and narrative skills. The above results can be explained in the following aspects.

First, in the early 1980s, Krashen (1981) proposed the language Input Hypothesis as the core theoretical framework in his language monitoring model, advocating that language acquisition requires understanding understandable inputs slightly higher than the existing level (the *i* + 1 model). This theory emphasizes that effective input needs to meet two conditions: one is sufficient language exposure, and the other is that the input content needs to be understandable and contain an *i* + 1 gradient structure.

According to Krashen’s language monitoring model and its core input hypothesis, the positive correlation between the duration of interactive reading intervention and the improvement of children’s narrative ability can be reasonably explained. This theory emphasizes that learners need to achieve language acquisition by being exposed to “*i* + 1” comprehensible inputs that are slightly higher than the existing level. In continuous intervention, young children are repeatedly exposed to texts that integrate known language (*i*) with new language elements (+1). This progressive input not only consolidly consoles basic abilities but also stimulates their interest in exploring new vocabulary, grammar and expressions. Especially for the development of narrative ability, the “*i* + 1” input is crucial because it involves the integration of language forms with plot events and emotional evaluations, as well as the ability to tell stories appropriately according to task requirements. With the increase of intervention duration, the narratives spontaneously generated by young children in natural conversations show significant improvements in terms of length, complexity and diversity (Umiker-Sebeok, 1979). Studies show that extending the duration of interactive reading intervention can significantly promote the development of narrative ability in young children, which is highly consistent with the core mechanism of Krashen’s language input hypothesis. According to the “*i* + 1” theory, continuous intervention provides a progressive language input environment for young children: by extending the exposure time, children constantly obtain compound texts containing known language (*i*) and new language elements (+1). This moderately challenging input not only consolidly consoles the existing language foundation but also continuously stimulates the desire to explore new vocabulary and grammatical structures. In the intervention practice, the dual guarantee mechanism of input quantity and quality has been effectively operated: With the extension of the intervention period, the amount of children’s language exposure increases exponentially, and by designing strategies such as narrative dialogues involving mothers, open-ended questions, and extensible feedback, it is ensured that the input always maintains an “*i* + 1” gradient (Sénéchal, 1997). Studies show that continuously strengthening language exposure and supplementing it with systematic narrative guidance can significantly enhance children’s dual abilities in language expression and narrative construction.

Second, educational theory points out that the cognitive development of young children is often based on the dual foundation of repeated practice and systematic training. Long periods of interactive reading also help establish a child’s narrative framework and structure. As stated by Sénéchal (1997), through repeated reading and questioning, young children are able to better understand and remember new vocabulary, which is very helpful for building complex narrative structures. During the continuous process of parent–child reading together, children gradually deepen their cognitive construction of the plot thread, character relationships and narrative logic through repeated exposure to texts on the same theme or various types of story carriers.

This can be argued for in pedagogy’s “progressive learning theory,” which is a training process that allows for continuous learning from input streams and growth over time, while retaining previously acquired knowledge (Karn et al., 2021). This tells us that learning should constantly contact relevant contents to consolidate achievements. Vretudaki et al. (2023) found in their study that through repeated reading and questioning, young children are able to better understand story structure and make more in-depth comments based on it. Schetz et al. (2000) also found in their study that interactive book reading and story retelling methods in small groups had positive effects on story comprehension and narrative skill development compared to large groups. Horst et al. (2011) found that 3-year-olds who read the same storybook three times in a week showed that children in the repeat reading group performed very accurately on both immediate recall and long-term memory tasks, whereas children who listened to different stories performed accurately only on immediate recall during the last two sessions. This means that repeated reading can help young children remember new vocabulary better and help them form complex narrative structures.

Third, interactive reading stimulates brain development through continuous language interaction, with a focus on promoting the functional improvement of the Broca area (responsible for language generation) and the Wernicke area (responsible for language comprehension). These two brain regions are connected through neural pathways such as the arboid bundle/superior longitudinal bundle, and there are significant differences in the maturity of their connections between the children and adult groups. And the efficient connection of these two regions is essential for language processing (Schmithorst et al., 2007). Scheinost et al. (2022) in his study showed that functional connectivity between Broca’s area and Wernicke’s area develops with age, which supports developmental changes in the brain in language processing. Schmithorst and Holland (2006) also confirmed in their study that effective connections between Broca’s area and Wernicke’s area increase with age, and Romeo et al. (2018) also found that children who had more conversation rounds with adults had stronger activation in Broca’s area. This enhanced connection contributes to improved narrative comprehension and expression (Schmithorst and Holland, 2006).

## 5 Limitations and future directions

However, this article still has limitations. First, inconsistencies across the included studies regarding national contexts, children’s environmental backgrounds, and narrative ability assessment tools may have contributed to variations in the results. Second, this study did not examine the moderating effects of factors such as individual differences among children and parental co-reading styles on the relationship between interactive shared reading and children’s narrative development.

Future research could address the following directions. First, future studies could include infants and toddlers aged 0–3 years to conduct comparative analyses of how interactive reading impacts children at different developmental stages. Second, research could further investigate the effect sizes of interactive shared reading on other developmental domains, such as executive function and emotional development.

## 6 Conclusion

This study employed a meta-analytic approach to evaluate the effectiveness of interactive reading interventions on the development of narrative skills in young children, as well as to examine the moderating effects of three key variables. The findings are summarized as follows: Interactive reading had a moderate and positive effect on children’s narrative skills (*g* = 0.425), and the intervention outcomes were significantly influenced by several moderator variables. The duration of the intervention significantly moderated the effect. Interventions lasting more than 17 weeks yielded the most substantial benefits for children’s narrative development. The type of participant involved in the intervention also had a significant moderating effect. Peer participation in interactive reading was found to have a more pronounced impact on children’s narrative skills compared to participation by researchers, teachers, or parents. Interactive reading was particularly effective for children aged 4–5 years, with this age group showing the greatest gains in narrative ability.

Through this research, parents and teachers can select the most appropriate interactive content based on children’s ages. By setting reasonable intervention cycles and actively guiding different objects such as peers and parents to participate, they can maximize the improvement of children’s narrative expression and comprehension abilities. This particularly has direct guiding value for early language intervention, children’s reading and writing assistance, and family reading guidance, promoting the development of children’s language literacy.

## Data Availability

The original contributions presented in the study are included in the article/supplementary material, further inquiries can be directed to the corresponding author.
